# Expression and purification of polioviral proteins in *E. coli*, and production of antisera as reagents for immunological assays

**DOI:** 10.1016/j.pep.2016.08.014

**Published:** 2016-12

**Authors:** Madala Uma, P.P. Rao, K. Nagalekshmi, N.R. Hegde

**Affiliations:** Ella Foundation, Genome Valley, Turkapally, Shameerpet Mandal, Hyderabad, 500078, India

**Keywords:** Poliovirus, Protein expression, Autoinduction, Immunoassays

## Abstract

Poliomyelitis, caused by poliovirus, is on the verge of eradication, and the world is preparing to shift from live to inactivated polio vaccine. In view of the requirement of non-infectious reagents, especially protein antigens, for surveillance during the final phase of poliovirus eradication, we have attempted to generate reagents that may be of use for the development of diagnostic tests. Polioviral proteins VP0, VP3, VP1, and 3AB were expressed in *Escherichia coli* using the autoinduction system, purified, and the proteins were used to raise antisera in rabbits. All antisera detected all three serotypes of PV from infected cell lysates in enzyme-linked immunosorbent assay, immunofluorescence and western blotting.

## Highlights

• Poliovirus structural and non-structural proteins were expressed in *E. coli* using autoinduction.
• Purified proteins were used for raising antibodies.
• Antisera raised could detect antigens in virus-infected cells by different immunoassays.
• Antisera could detect both cleaved and uncleaved as well as native and denatured viral proteins.
• These reagents may be useful in vaccine manufacturing and seromonitoring in the future.

## 1 Introduction

Polio is an acute viral infectious disease of humans, and is caused by poliovirus (PV). PV is a single-stranded positive sense RNA virus belonging to the genus *Enterovirus* of the family *Picornaviridae*. PV is classified into three serologically distinct types, PV1, PV2, and PV3, each with slight differences in capsid structure, which are differentially recognized in virus neutralization assays. The PV genome is about 7500 nucleotides long, and consists of a single open reading frame flanked by a 5′ non-translated region (NTR), which includes the internal ribosome entry site (IRES), 3′NTR, and a polyA tail. The small viral protein VPg is covalently linked to the 5′ end of the genome. Translation of the viral genome results in the synthesis of a polyprotein, which is cleaved by viral proteases into individual viral proteins, including (a) three structural proteins VP1, VP3 and VP0, the latter of which is further processed into VP4 and VP2 during virion morphogenesis, and (b) seven non-structural proteins (NSP's: 2A, 2B, 2C, 3A, 3B, protease 3C, and RNA-dependent RNA polymerase 3D). The 2A and 3C proteins are responsible for the proteolytic cleavage of the polyprotein [1], [2].

Three major antigenic sites with neutralization epitopes are present on the surface of PV and the location of the antigenic sites differs among serotypes. Antigenic site 1 (residues 89–100 of VP1) is present in PV2 and 3, antigenic site 2a (residues 220–222 of VP1) is present in PV1, antigenic site 2b (residues 164–172 of VP2) is present in PV1 and 3, antigenic site 3a (residues 286–290 of VP1) is present on PV3, whereas antigenic site 3b (residues 58–60, 70, 71, 77, 79 of VP3) is present in PV1 and 3 [3], [4]. Cross-reactive antibodies that bind to PV receptor binding site and neutralize PV1 and 2 have also been reported [5]. Apart from neutralizing antibodies, non-neutralizing cross-reactive antibodies raised against denatured antigen can recognize all three serotypes of PV [6], [7]. In addition to antibodies to structural proteins, infected hosts also produce antibodies against non-structural proteins [8], [9]; however, antibodies against NSP's of PV are more likely to be serotype-independent, compared to antibodies to structural proteins.

Seromonitoring of mass vaccination campaigns requires demonstration of high antibody titers against structural proteins, especially VP1. On the other hand, demonstration of anti-NSP antibodies in the sera is an indication of infection. The 3AB protein is absent in purified inactivated vaccines, and antibodies against NSP indicate circulation of PV in a population. Immunological assays developed based on recombinant PV proteins could therefore be useful for demonstration of freedom from infection status after cessation of oral polio vaccine.

The inducible T7 expression system is widely used for expressing foreign genes in *E. coli* [10], [11]. In general, the cells are grown to increase the mass before inducing protein expression. Alternatively, an autoinduction system [12], [13] can be employed, where gene expression will be induced once glucose is completely used up, and the cell switches to lactose as the sole source of carbon. This method is advantageous because there is no need to monitor either the cell growth or the time of induction during autoinduction. In the current study, the autoinduction system was used to express *VP0, VP3, VP1* and *3AB* genes of PV1 in *E. coli*, and antisera raised using the recombinant proteins was tested for their suitability to detect different serotypes of PV in different immunoassays.

## 2 Materials and methods

### 2.1 Plasmids and *Escherichia coli (E. coli)* strains

The pRSET B (Invitrogen, Bengaluru, India) plasmid was used for cloning and expression of genes of interest under the control of the T7 ϕ10 promoter. The *E. coli* DH5α strain (Invitrogen) was used for cloning of genes, while *E. coli* BL21(DE3) strain (Invitrogen) was used for protein expression.

The pVS(1)IC-O(T) plasmid (kind gift from Dr. Akio Nomoto, Tokyo University, Japan), which contains the full length PV1 genome under the control of SV40 promoter [14], was used as an infectious plasmid to produce PV1.

### 2.2 Viruses and cells

CV1 (ATCC-CCL-70) is a fibroblast cell line derived from male African green monkey kidney. CV1 cells are susceptible for poliovirus infection [15].

Sabin strains of PV1, PV2, and PV3 were obtained from Bharat Biotech International Limited, Hyderabad, India. Recombinant PV1 (rPV1) was generated using plasmid pVS(1)IC-O(T) by transfecting CV1 cells (ATCC-CCL-70). For making lysates, all virus infections were carried out at 0.1 multiplicity of infection (MOI), whereas for immunofluorescence, MOI of 0.01 (PV1) or 0.05 (PV2 and PV3) were used. All experiments were carried out in biosafety level II laboratory, following appropriate protocols and procedures.

### 2.3 Bacterial culture media

The following bacterial culture media were used in the present study:

LB-SP medium: 1% soy peptone, 0.5% yeast extract, 1% sodium chloride.

ZYP-0.8G-SP medium: 1% soy peptone, 0.5% yeast extract, 50 mM Na_2_HPO_4_, 50 mM KH_2_PO_4_, 25 mM (NH_4_)_2_SO4, 2 mM MgSO_4_ and 0.8% glucose.

ZYP-5052-SP medium for autoinduction: 1% soy peptone, 0.5% yeast extract, 50 mM Na_2_HPO_4_, 50 mM KH_2_PO_4_, 25 mM (NH_4_)_2_SO_4_, 2 mM MgSO_4,_ 0.5% glycerol, 0.05% glucose and 0.2% α-lactose.

### 2.4 Cloning of polioviral genes

*VP0, VP3, VP1* and *3AB* genes of Sabin PV1 (AY184219.1) were amplified by RT-PCR using gene-specific primers with flanking restriction sites *Eco*RI and *Hin*dIII (Table 1) and cloned into pRSET B in frame with the N-terminal His-tag, using standard procedures [16].

### 2.5 Transformation and expression of genes

The recombinant plasmids pPV1VP0, pPV1VP3, pPV1VP1, pPV13AB as well as control pRSET B (not containing any insert) were transformed into chemically competent BL21(DE3) cells [17]. Transformed cells were grown in one mL of LB-SP broth. After 1 h of incubation at 37 °C, 100 μL of the culture was spread on LB-SP agar containing 2% glucose with 100 μg/mL of ampicillin for selection, and incubated at 37 °C overnight. From each transformed plate a few colonies were transferred to 2 mL of ZYP-0.8G medium containing ampicillin (100 μg/mL) and incubated in a rotary shaker at 200 rpm at 37 °C. After 6–8 h, 100 μL of the culture was inoculated into 200 mL of ZYP-5052 autoinduction medium with antibiotic, and further grown in culture with shaking at 37 °C overnight. The resulting culture was centrifuged (1500*g* for 10 min), and the cells were used for analysis.

### 2.6 SDS-PAGE and western blotting

One mL of autoinduced culture was pelleted and resuspended in 1X reducing SDS-PAGE loading buffer, boiled at 95 °C for 10 min, subjected to 12% denaturing SDS-PAGE and stained with Coomassie brilliant blue R-250. Gels were blotted to PVDF membranes for western blot analysis. The membrane was blocked with 5% w/v skimmed milk powder (SMP) in phosphate buffered saline, pH 7.2 (PBS), overnight at 4 °C. The membrane was incubated with anti-His monoclonal antibodies (Sigma-Aldrich, Bengaluru, India) at a dilution of 1:15,000 in 3% SMP in PBS at 37 °C for 1 h, washed three times with PBS containing 0.05% Tween-20 (PBST), followed by incubation with a 1:10,000 dilution of horseradish peroxidase (HRP) conjugated anti-mouse IgG secondary antibody in 3% SMP at 37 °C for 1 h. The membranes were washed three times with PBST, and once with PBS, then incubated with ECL reagent (Thermo Scientific, Bengaluru, India), and His-tagged proteins were detected using X-ray film.

### 2.7 Protein solubility

Ten mL of autoinduced cultures were pelleted and resuspended in 10 mL of 50 mM Tris (pH 8.0) buffer containing 25% sucrose, 0.5% Triton X-100, and 1 mM phenyl methyl sulfonyl fluoride (PMSF). Cells were lysed by sonication (10 cycles of 15 s pulse with intervals of 30 s each, at 60% amplitude) on ice, and the lysate was pelleted by centrifugation at 16,000*g* for 10 min. The supernatant and pellet were collected separately and analyzed on reducing SDS-PAGE.

### 2.8 His-tag based nickel-nitriloacetic acid (Ni-NTA) protein purification

#### 2.8.1 Purification of soluble proteins

Sonicated lysate was clarified by centrifugation at 16,000*g* for 5 min, and used to load a Ni-NTA column pre-equilibrated with 50 mM Tris-HCl, 0.3 M NaCl, 20 mM imidazole, pH 8.0 (soluble protein equilibration buffer). The column was washed with 50 mM Tris–HCl, 0.3 M NaCl, 20 mM imidazole, pH 8.0 (soluble protein wash buffer), until the OD at A_280_ reached zero, and the column-bound protein was eluted with 50 mM Tris–HCl, 0.3 M NaCl, 250 mM imidazole, pH 8.0 (soluble protein elution buffer). The elution fractions were analyzed on reducing SDS-PAGE, and fractions with the presumed desired protein were pooled and dialyzed against 100 mM Tris, pH 8.0 overnight at 4 °C. Protein concentration was estimated by BCA protein assay kit (Thermo Scientific) according to the manufacturer's instructions.

#### 2.8.2 Purification of proteins from inclusion bodies

After sonication, the cell pellet was dissolved in 10 mL of 50mMTris-HCl, 8 M urea, pH 8.0 (denatured protein equilibration buffer), clarified by centrifugation at 16,000*g* for 5 min, and the supernatant was loaded onto a Ni-NTA column pre-equilibrated with denatured protein equilibration buffer. The column was washed with 50 mM Tris-HCl, 8 M urea, 20 mM imidazole, pH 8.0 (denatured protein wash buffer) until OD at A_280_ reached zero, and column bound proteins were eluted with 50 mMTris–HCl, 8 M urea, 250 mM imidazole, pH 8.0 (denatured protein elution buffer) and collected fractions were analyzed by reducing SDS-PAGE. Fractions with presumed desired protein were pooled, and urea was removed by step dialysis through a graded series of urea (6 M, 4 M, and 2 M) in 100 mM Tris, pH 8.0, for 2 h each, followed by a final dialysis against 100 mM Tris, pH 8.0, overnight, all at 4 °C. Protein concentration was estimated by BCA protein assay kit (Thermo Scientific) according to the manufacturer's instructions.

### 2.9 Production of hyperimmune sera

Animal experiments were conducted with permission from the Institutional Animal Ethics Committee as per the Guidelines of the Committee for the Purpose of Control and Supervision of Experiments in Animals, under the Breeding of and Experiments on Animals (Control and Supervision) Rules, 1998 as amended in 2001 and 2006, Government of India.

Five hundred micrograms of the purified recombinant proteins (PV1-VP0, -VP3, -VP1 and -3AB) each were mixed individually with equal volume of Freund's incomplete adjuvant (FIA) to make an emulsion and injected into 2-month-old New Zealand white rabbits subcutaneously. On day 28, the animals were boosted with 250 μg of respective protein emulsions in FIA. On day 35, sera were tested for the ability to detect PV-specific proteins by western blotting. A second booster of 250 μg of respective protein emulsions in FIA was administered on day 56, and sera obtained at day 66 were stored at −20 °C.

### 2.10 Immunological reactivity of antibodies

The sera were tested for their ability to detect three serotypes of PV as well as rPV1. CV1 cells were infected with PV1, PV2, or PV3 viruses, or transfected with pVS(1)IC-O(T) plasmid. At 30% cytopathic effect, cell lysates were prepared by freeze-thawing in PBS, and used for western blotting and ELISA. Similarly, the ability of the antisera to detect the three serotypes of PV by immunofluorescence assay was tested on CV1 cells infected with PV1, PV2, or PV3 viruses.

#### 2.10.1 Enzyme-linked immunosorbent assay

ELISA plates were coated with PV1, PV2, or PV3 infected or uninfected CV1cell lysates (100 μg of total protein/well) or recombinant proteins (100 ng/well) overnight at 4 °C. The plates were blocked with 3% SMP in PBS for 1 h at 37 °C. Different polyclonal sera at various dilutions (1:200 to 1:3200) in 1% SMP in PBS were added and incubated at 37 °C for 1 h, washed three times with PBST, followed by addition of HRP-conjugated anti-rabbit IgG (Sigma-Aldrich) at 1:5000 dilution in 1% SMP at 37 °C for 1 h. The plate was washed three times with PBST and once with PBS, and then developed using *o*-phenylene diamine (OPD) substrate. The reaction was stopped by addition of 50 μL of 3 M sulphuric acid per well and the OD was read at 490 nm. The ELISA data was analyzed using one-way analysis of variance (GraphPad Prism). The p values were calculated by comparing lysates from control CV1 cells to lysates from CV1 cells infected with PV1, PV2 or PV3, using Bonferroni's multiple comparison test.

#### 2.10.2 Western blotting

Lysates of PV1-, PV2-, or PV3-infected CV1 cells, or extracts of pVS(1)IC-O(T) plasmid- or mock transfected CV1 cells were subjected to reducing SDS-PAGE and transferred to a PVDF membrane. Anti-PV1-VP0, -VP3, -VP1or -3AB rabbit sera were used as primary antibody (1:1000) and HRP-conjugated anti-rabbit IgG (Sigma-Aldrich) (1:5000) was used as secondary antibody to develop western blot using ECL reagent (Thermo Scientific) to detect respective PV proteins.

#### 2.10.3 Immunofluorescence

CV1 cells were infected with PV1 (0.01MOI), PV2 (0.05 MOI), or PV3 (0.05MOI), and incubated at 37 °C for 18 h. The cells were fixed with methanol: acetone (1:1) mixture at room temperature for 3 min, and washed twice with PBS. Rabbit anti-VP0, -VP3, -VP1 or -3AB polyclonal sera were added to each well at 1:200 dilution, and incubated at 37 °C for 1 h, and the plates were washed three times with PBS. Then, fluorescein isothiocyanate (FITC)-conjugated anti-rabbit IgG (Sigma-Aldrich) (1:500 dilution) was added, before incubation at 37 °C for 1 h, and two washes with PBS. The cells were then observed under a fluorescent microscope.

## 3 Results and discussion

### 3.1 Cloning PV genes, and expression and purification of PV proteins

**Fig. 1:**
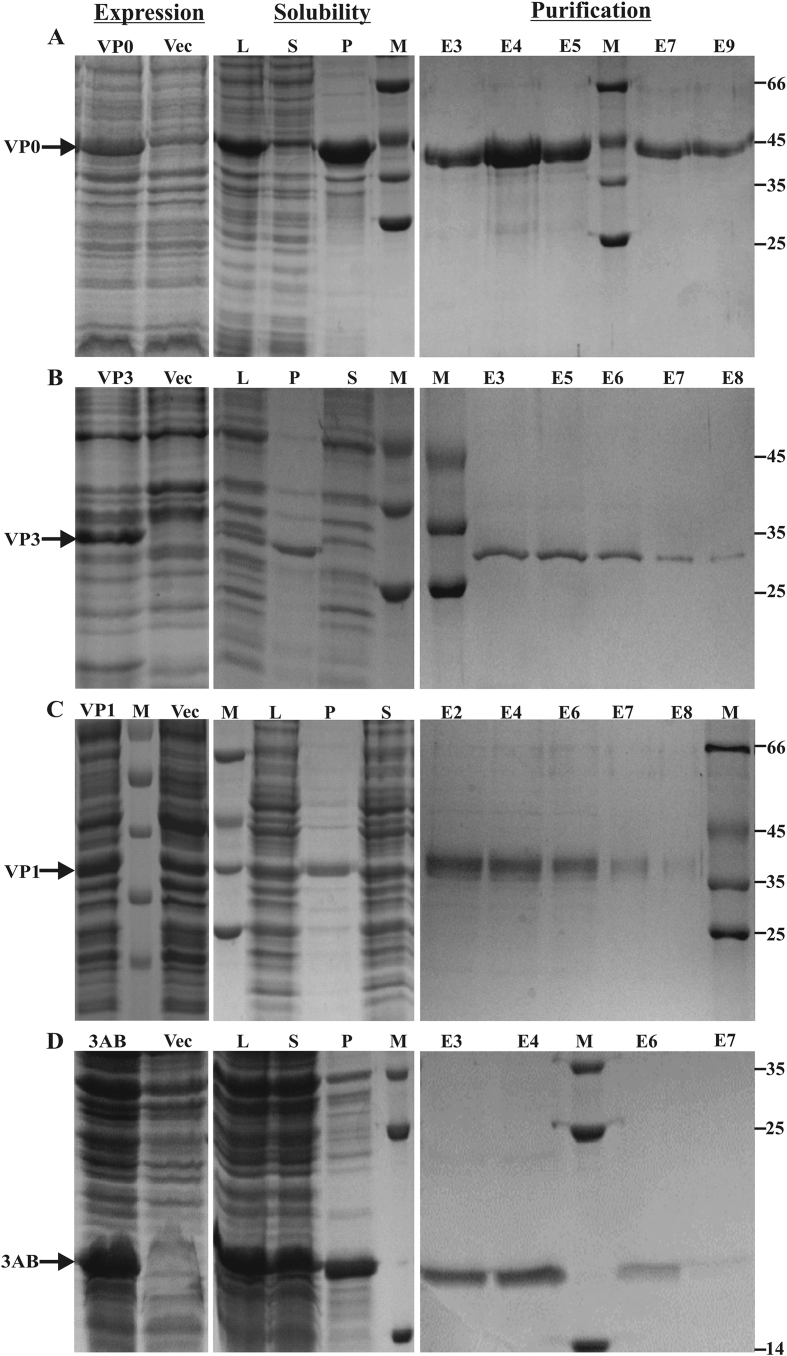
**Expression and purification of PV proteins in *E. coli***: Induced proteins were subjected to reducing SDS-PAGE and visualized by Coomassie blue staining. Specific protein expression from each gene can be observed by comparison with pRSET B *E. coli* lysate. (A) PV1-VP0, (B) PV1-VP3, (C) PV1-VP1, (D) PV1-3AB gene expression, solubility and purification using Ni-NTA chromatography. Vec: pRSET B vector control, L: total cell lysate after induction, S: supernatant after sonication, P: pellet after sonication, E: different elution fractions after purification, M: molecular weight marker standards (Sigma-Aldrich) containing bovine serum albumin – A7517 (∼66 kDa), chicken ovalbumin – A7642 (∼45 kDa), glyceraldehyde 3-phosphate dehydrogenase (GAPDH) – G5262 (∼35 kDa), bovine pancreatic trypsinogen – T9011 (∼24 kDa), and bovine α-lactalbumin – L6385 (∼14 kDa).

**Fig. 2:**
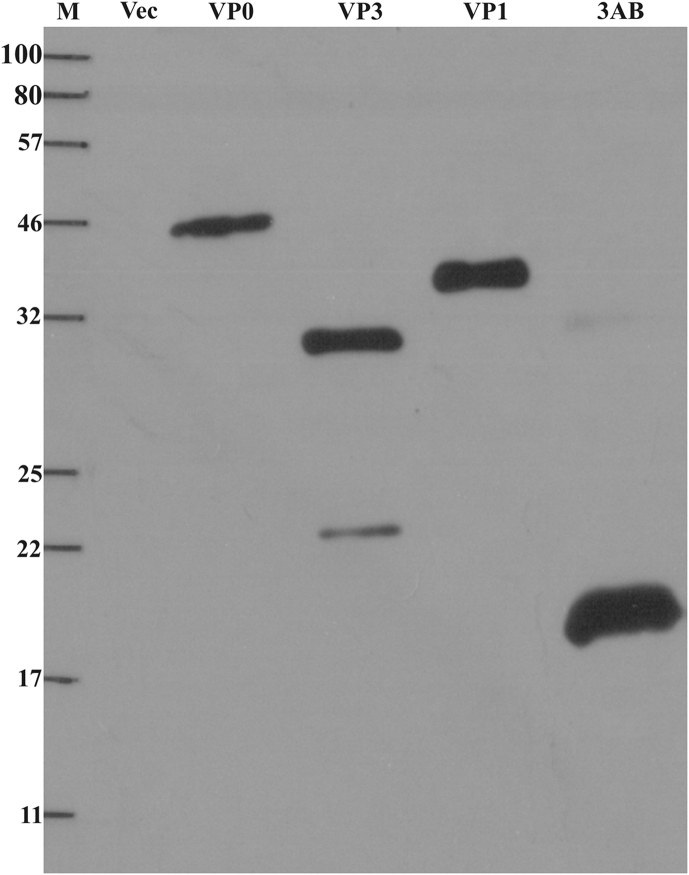
**Confirmation of tagged proteins**: Five μL of each of the column purified proteins were subjected to reducing SDS-PAGE, blotted, and the His-tagged proteins were detected using anti-His antibodies. Vec: pRSET B vector control, M: molecular weight marker (Color Prestained Protein Standard, NEB, Cat. no-P7712L).

**Fig. 3:**
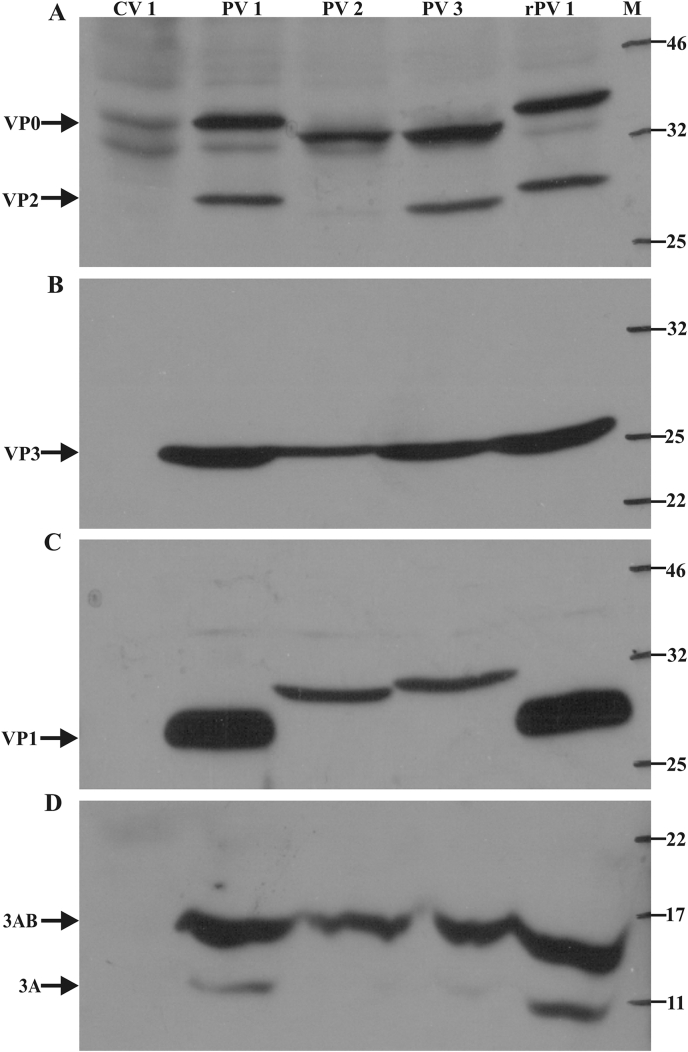
**Detection of PV proteins in infected cells by polyclonal sera**: CV1 cells were separately infected with the three serotypes of PV, or left uninfected. The cells were harvested 48 h post-infection and lysates were subjected to reducing SDS-PAGE and western blotting using rabbit polyclonal antibodies (anti-PV1-VP0 sera for panel-A, anti-PV1-VP3 sera for panel-B, anti-PV1-VP1sera for panel-C and anti-PV1-3AB sera for panel-D). rPV1 lysate was prepared by transfecting CV1 cells with pVS(1)IC-O(T) plasmid. M: molecular weight marker (Color Prestained Protein Standard, NEB, Cat.no-P7712L).

The amplified PCR products for PV1 *VP0, VP3, VP1* and *3AB* genes were cloned into pRSET B plasmid, and positive clones (pPV1VP0, pPV1VP3, pPV1VP1, pPV13AB) were confirmed by restriction digestion and nucleotide sequencing. All the genes were expressed individually in *E. coli* BL21(DE3) using autoinduction [13]. Expression of the proteins in the induced cultures was assessed by visualization of an additional protein band in the induced cultures compared to controls on reducing SDS-PAGE (Fig. 1; panel A for VP0, panel B for VP3, panel C for VP1 and panel D for 3AB). Additional bands corresponding to VP0 (42 kDa), VP3 (31 kDa), VP1 (38 kDa) and 3AB (17 kDa) were also observed compared to the controls. The molecular weights of all the recombinant proteins were approximately 5 kDa greater than the native viral proteins due to fusion of N-terminal coding sequences from the vector (His tag, T7 gene10 leader, Xpress epitope tag and enterokinase recognition site). Proteins VP0, VP3 and VP1 were present in the insoluble fraction (inclusion bodies; pellet fraction with each of the proteins in Fig. 1). This was not surprising since PV structure is formed by 60 copies each of VP0, VP1 and VP3, and these proteins may not fold properly when expressed independently, leading to inclusion body formation [18]. These proteins were denatured with urea before applying for purification on Ni-NTA column. In the case of 3AB, the protein was present in both soluble and insoluble fractions (Fig 1, panel D, middle). The presence of 3AB in the insoluble fraction is not unexpected as the protein has been shown to be membrane-associated in both virus-infected eukaryotic cells [19] and when expressed in *E. coli* [20]. However, for the ease of purification, only soluble fraction of 3AB was used. Purification on Ni-NTA column resulted in more than 90% purity for all the expressed proteins. The final purified protein yields obtained when cultured in shake flasks were 36 μg (VP0), 16 μg (VP3), 28 μg (VP1), and 19 μg (3AB) per mL of culture, respectively. When dialyzed samples of pooled elution fractions were subjected to western blotting, anti-His antibodies could detect specific bands of all the proteins, except in the case of VP3, where an additional smaller band of the protein was observed (Fig. 2). This latter band could be a degradation product of VP3 protein where the C-terminus was lost, since the recombinant protein was generated using an N-terminal His-tag. The slight discrepancy in the apparent molecular weight of 3AB between Fig. 2, Fig. 3 could be due to differential migration of unstained versus pre-stained molecular size markers, respectively.

### 3.2 Reactivity of hyperimmune sera

**Fig. 4:**
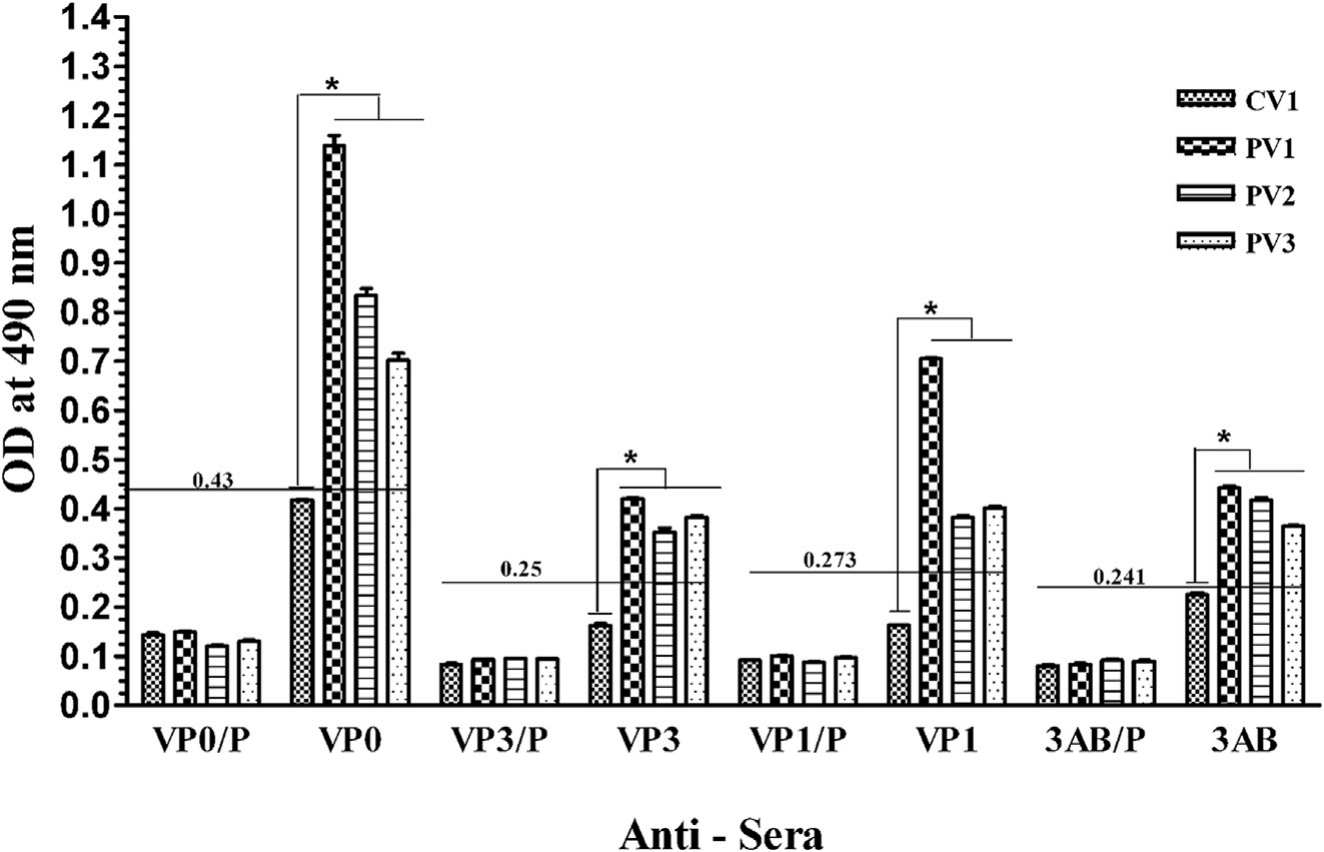
**Testing of polyclonal sera in ELISA**: The ELISA plates were coated with CV1 cell lysates infected with PV1, PV2, or PV3, or uninfected cell lysates. Different polyclonal sera (against VP0, VP3, VP1, and 3AB) along with their pre-immune sera (denoted ‘P’) were used as primary antibody followed by HRP-conjugated secondary antibody for detection. The error bar shows the standard deviation of duplicate samples. Cut-off to declare positivity (horizontal line) was taken as three times the average of pre-immune sera. The p values were calculated by comparing control CV1 lysate with PV1, PV2 or PV3 lysates using Bonferroni's multiple comparison test.(*P value < 0.001).

The anti-PV1-VP0 sera could detect both VP0 (37 kDa) and VP2 (30 kDa) proteins in western blot analysis of lysates from cells infected with all three serotypes of PV, or transfected to obtain rPV1 (Fig. 3, panel-A). However, only a faint band of VP2 was observed with PV2 lysate. Similarly, the anti-PV1-3AB sera could detect 3AB (12 kDa) in all the samples, except lysates from cells alone (Fig. 3, panel-D). In case of homologous virus (PV1 and rPV1) the sera could detect 3A protein also. These results indicate that both the sera could detect uncleaved and cleaved proteins in case of proteins of homologous virus, whereas detection of cleaved protein varied in case of proteins of heterologous viruses. The anti-PV1-VP3 sera could detect VP3 (26 kDa) from all the serotypes of PV as well rPV1 (Fig. 3, panel-B). In the case of anti-PV1-VP1 sera, VP1 (33 kDa) of all serotypes of PV could be detected; however, the signal was better with PV1 (both PV1 and rPV1) as compared to that with PV2 and PV3 (Fig. 3, panel-C). It is worth noting that VP1 is a serotype-specific protein, although some level of cross-reactivity between different serotypes can be observed [7], [21]. This could be the reason for the observation of better signal with homotypic than the heterotypic VP1. Further, a difference was observed in the mobility (gel shifting) of VP0 and VP1 proteins of different serotypes of PV (Fig. 3, panels A and C). Similar differences in mobility of PV proteins have been reported earlier [22], [23]. The formula weight of the VP0 and VP1 proteins of all the serotypes is similar; however, they differ in charge, which may be contributing to the shifting of these proteins on SDS-PAGE. Gel shifting is a common phenomenon observed in highly acidic or basic proteins, as well as proteins with proline kinks, and among mutant proteins with one or two amino acid changes [24], [25], [26], [27]. By ELISA, hyperimmune sera could detect *E. coli* expressed recombinant proteins (Supplementary Information S. Fig. 1) as well as viral protein in the PV-infected cell lysates (Fig. 4, Supplementary Information S. Fig. 2). ELISA results were similar to western blot analysis, in that (a) all antisera could detect PV1, PV2, and PV3, (b) anti-VP1 antibodies reacted better with homologous virus (PV1) than with heterologous viruses (PV2 or PV3), and (c) anti-VP0 sera showed some background reactivity with CV1 cell lysate (Fig. 4; also compare with data in Fig. 3). Significant difference was observed with all polyclonal sera when OD values were compared between control (uninfected) CV1 cells and CV1 cells infected with PV1, PV2 or PV3 (P > 0.001).

**Fig. 5:**
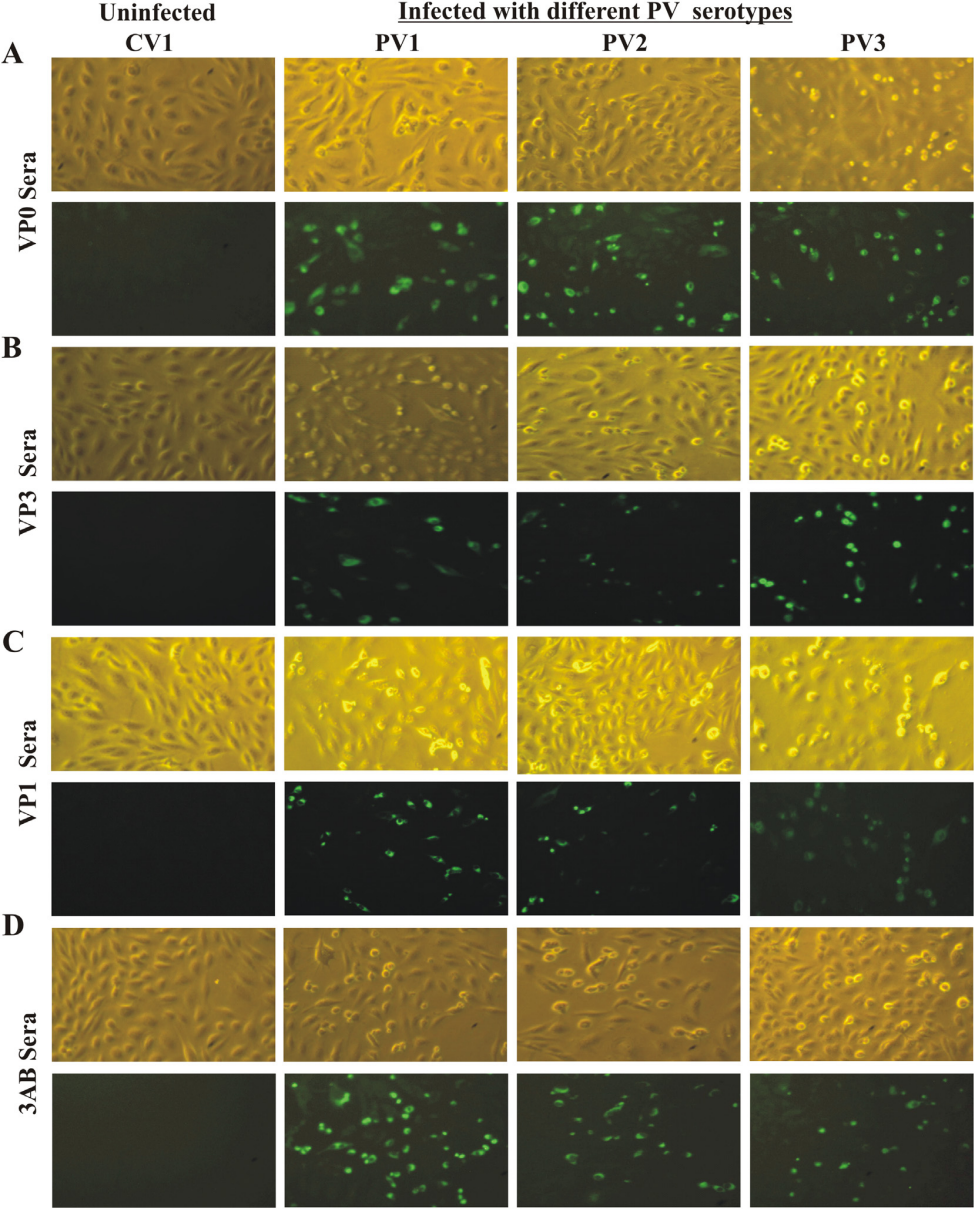
**Testing of polyclonal sera in immunofluorescence**: Cell monolayers were infected with PV1, PV2, or PV3, or left uninfected. Different polyclonal sera raised in rabbits (against VP0, VP3, VP1 and 3AB) were used as primary antibody followed by FITC-conjugated secondary antibody for detection.

As expected, all sera also showed signal in cells infected with all three serotypes of PV, and no signal was observed in uninfected cells by immunofluorescence assay (Fig. 5). Pre-immune sera did not show any background staining with cells infected with PV (data not shown). However, in contrast to the results with western blot and ELISA, no observable difference in fluorescence intensity among the serotypes could be detected. This could possibly be due to the lower sensitivity of the assay as well as potential higher background in infected cells.

Detection of PV proteins in western blot, ELISA and immunofluorescence indicate that the sera could detect both native and denatured proteins. Antibodies developed during this study can be used for vaccine identity test and probably also for potency testing, and we are currently exploring these applications with a vaccine manufacturer. It should be noted that extensive standardization in terms of production, purification, and quality control of the proteins is required to use them as validated reagents. Similarly, the polyclonal sera also need to be extensively characterized. In this context, characterized monoclonal antibodies may provide more standardized antibody reagents, although polyclonal antibodies cover multiple reactive sites on a protein.

## 4 Conclusions

Owing to the PV eradication initiative, the incidence of polio due to wild type virus is on the verge of approaching zero. In this transitory period, the oral, live attenuated vaccine is being replaced by the injectable, inactivated vaccine [28], [29]. As we move towards containment of live polioviruses followed by destruction of the stocks, protein- and not virus-based reagents are essential both for evaluation of immune responses as well as for surveillance studies to detect circulating vaccine-derived PV's. The reagents generated (both proteins and antisera) in this study are a step forward in the development of analytical assays useful for virus identity during vaccine manufacturing and also for development of immunoassays for monitoring the eradication program.

## Figures and Tables

**Table 1:** Primers used for amplification of PV genes.

Primer name	Sequence (5′–3′; enzyme sites in bold face)
PV1-VP0*Eco*RI F	ATAC**GAATTC**ATGGGTGCTCAGGTTTCATCACAGA
PV1- VP0*Hin*dIII R	TAAC**AAGCTT**TTATCACTGTAAGCGTGGCAGGGTAATGT
PV1-VP3*Eco*RIF	ATAC**GAATTC**GGCCTGCCGGTCATGAACAC
PV1-VP3*Hin*dIII R	TAAC**AAGCTT**TTATCACTGTGCTAGCGCTTTTTGCTCT
PV1- VP1*Eco*RIF	GGAT**GAATTC**GGGTTAGGTCAGATGCTTGAAA
PV1- VP1*Hin*dIIIR	TGGC**AAGCTT**TTATCAATATGTGGTCAGATCCTTGGTGGA
PV1- 3AB*Eco*RI F	TATC**GAATTC**GGACCACTCCAGTATAAAGAC
PV1- 3AB*Hin*dIII R	GCTC**AAGCTT**TTATCATTGTACCTTTGCTGTCCTAATG
