# Supplementary material for: Expression and purification of polioviral proteins in E. coli, and production of antisera as reagents for immunological assays
Source: Protein Expr Purif. 2016 Dec;128:115–22. doi: 10.1016/j.pep.2016.08.014 (PMC5040459; doi:10.1016/j.pep.2016.08.014)
Supplement: Supplementary file 1 [file mmc1.docx]

**Expression and purification of polio viral proteins in *E. coli*, and production of anti-sera as reagents for immunological assays**

Madala Uma., Rao P. P., Nagalekshmi K., Hegde N.R.*

Ella Foundation, Genome Valley, Turkapally, Shameerpet Mandal, Hyderabad-500075, India

*Corresponding author: Nagendra R Hegde

Ella Foundation

Genome Valley

Turkapally, Shameerpet Mandal

Hyderabad – 570078

Phone: +91-40-23480570

Fax: +91-40-23480571

E mail: [hegden@ellafoundation.org](mailto:hegden@ellafoundation.org)


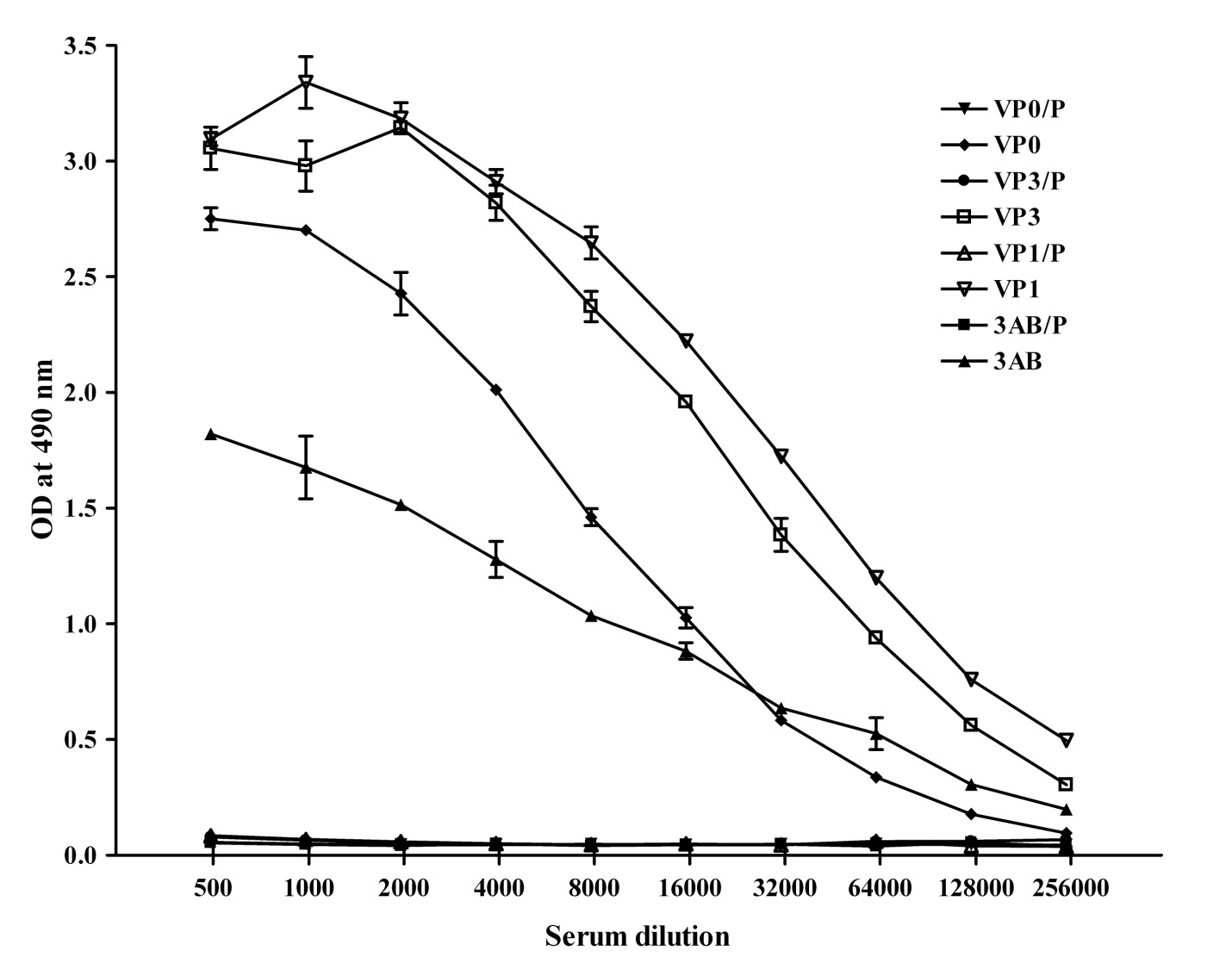


**S. Fig. 1. Testing of polyclonal sera with respective recombinant proteins in ELISA**: The ELISA plates were coated with 100ng of respective recombinant protein as antigen. Different polyclonal sera (against VP0, VP3, VP1, and 3AB) along with their pre-immune sera (denoted ‘P’) were used as primary antibody followed by HRP-conjugated secondary antibody for detection. The error bars show standard deviation of duplicate samples.


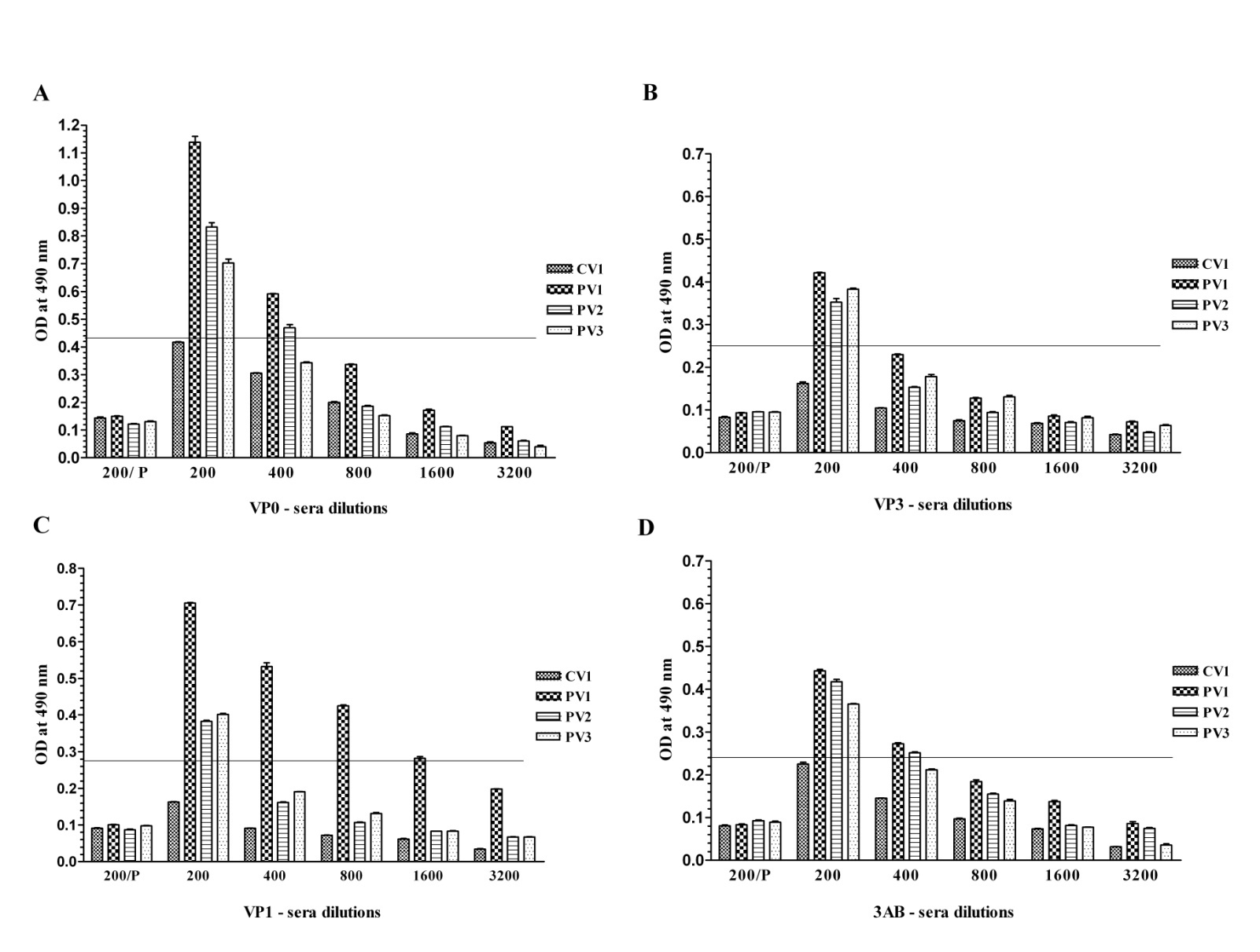


**S. Fig. 2. Titration of polyclonal serafor use in ELISA**: The ELISA plates were coated with CV1 cell lysates infected with PV1, PV2, or PV3, or uninfected cell lysates. Different polyclonal sera (against VP0, VP3, VP1, and 3AB) along with their pre-immune sera (denoted ‘P’) were used as primary antibody followed by HRP-conjugated secondary antibody for detection. The error bars show standard deviation of duplicate samples.
